# Incidence rate and distinctive characteristics of first episode psychosis during the COVID-19 pandemic: a multicenter observational study

**DOI:** 10.1038/s41598-022-26297-6

**Published:** 2022-12-21

**Authors:** Francesc Casanovas, Amira Trabsa, Daniel Bergé, Vanessa Sánchez-Gistau, Irene Moreno, Julia Sanchez, Itziar Montalvo, Meritxell Tost, Javier Labad, Victor Pérez-Solà, Anna Mané

**Affiliations:** 1 Institut de Neuropsiquiatria i Adiccions (INAD), Parc de Salut Mar, Barcelona, Spain; 2 Hospital del Mar Medical Research Institute (IMIM), Barcelona, Spain; 3 Department of Medicine and Life Sciences (MELIS), Universitat Pompeu Fabra, Barcelona, Spain; 4 Centro de Investigación Biomédica en Red, Área de Salud Mental (CIBERSAM), Madrid, Spain; 5 Hospital Universitari Institut Pere Mata, IISPV, Universitat Rovira i Virgili, Reus, Spain; 6 Department of Mental Health, Hospital Universitari Parc Taulí, I3PT, Sabadell, Spain; 7 Department of Mental Health, Consorci Sanitari del Maresme, Mataró, Spain

**Keywords:** Medical research, Risk factors

## Abstract

The COVID-19 pandemic has affected the mental health of people around the world. However, its impact on first-episode psychosis (FEP) remains unclear. The aim of this study was to determine the incidence rate (IR) and the clinical and sociodemographic characteristics of patients who developed FEP during the nine-month period following the COVID-19 outbreak in Spain and to compare these data to the corresponding period in the previous year. We included all patients (n = 220) treated for the first time during these two time periods at three FEP programs in Spain. The IR was 0.42/100,000 person-years during the pandemic vs. 0.54/100,000 in the prior year (p = 0.057). Compared to prior year, women accounted for a significantly higher proportion of FEP patients (46.3% vs. 28%; p = 0.005) during the COVID-19 period. This association was significant on the logistic regression analysis (odds ratio, female: 2.12 [confidence interval 1.17–3.82]; p = 0.014). These data reveal a non-significant trend towards a lower incidence of FEP during the pandemic period. Female sex was associated with a greater risk of developing FEP during the pandemic period, perhaps due to differences between males and females in the susceptibility and expression of psychosis. The findings of this study contribute to a better understanding of stress-related disorders.

## Introduction

COVID-19 is an infectious disease caused by a type of coronavirus (SARS-CoV-2). Since the first case was detected in December 2019 in Wuhan, China, the disease has spread rapidly around the world, with more than 83 million people infected and 1.88 million deaths by the end of 2020^1^. The respiratory manifestations of COVID-19 ranges from asymptomatic to a mild upper respiratory infection or even pneumonia, potentially leading to severe acute respiratory syndrome (SARS)^2^. Infection with SARS-CoV-2 can cause various extrapulmonary symptoms that appear to be related to a hyperinflammatory state induced by dysregulation of the innate immune system^3^. People with COVID-19 can also present neuropsychiatric conditions such as delirium, anxiety, depression, psychosis, and post-traumatic stress disorder (PTSD) due to the effects of the virus on the central nervous system, hyperinflammation, and to the psychosocial context of the infection^4^. Early in the pandemic, it was predicted that these psychosocial stressors would affect the entire population^5–7^. Those predictions seem to have been correct, as the scientific evidence points to a growing impact on global mental health^8^. Some of the factors underlying these stressors are related to the disease itself, such as the fear of getting infected or grief over the death of loved ones. Secondary factors include government-imposed restrictions for epidemiologic control, such as lockdowns and social distancing mandates. The economic impact of the pandemic, which has caused job losses for many people, is another important stressor. Many of these stressors were particularly acute in Spain, which established some of the harshest lockdown measures in terms of the severity and duration of the lockdown. Moreover, the cultural characteristics of the society (e.g., an active social life with frequent physical contact) may have negatively influenced people’s ability to adapt to the situation^9^. The most severe restrictions were imposed between March and June 2020 when the Spanish public health system was overwhelmed by the high infection and mortality rates, which were among the highest in Europe^10^. After two more waves of infections, the total number of people infected in Spain in the year 2020 was 1.93 million, with 50,000 deaths^1^.

Recent studies have found higher levels of anxiety and depression symptoms in the general population during the first^11^ and second waves^12^ of the pandemic. Longitudinal studies have shown that these symptoms did not disappear even after lockdown measures were relaxed^13^, suggesting that these stressors continued to negatively affect mental health several months after the onset of the pandemic.

A surveillance study found that new onset psychosis is one of the neuropsychiatric symptoms associated with SARS-CoV-2 infection^14^. However, the impact of the pandemic on the incidence of newly-diagnosed psychosis in people who have not been infected with SARS-CoV-2 remains unclear. This potential association is relevant and warrants study given that psychosocial stress is a known risk factor for first-episode psychosis (FEP) in vulnerable people^15,16^. Some of the environmental factors linked to a greater risk of developing psychosis, such as migration and urbanicity, involve a disruption in normal social functioning^17^. The COVID-19 pandemic is a unique period of time that offers us the opportunity to investigate an objective stressful event, similar to other historical circumstances such as wars or natural disasters^17^).

Brief psychotic disorder is classically associated with acute stressors, as its DSM-V specifier indicates. Brief psychotic disorder is characterized by the presence of psychosis (delusions, hallucinations, disorganized speech and behavior) lasting for less than one month followed by total recovery of the previous level of functioning^18^. After the first few months of the pandemic, some reports suggested a rise in the number of brief psychotic disorder cases. For example, researchers in Milan, Italy described a series of cases in which the disorder tended to develop at slightly older ages than usual and was accompanied by suicidal ideation^19^. Similar findings were observed in Spain^20^. Later, both of those groups published new studies with more clinical details. The Spanish group evaluated the characteristics of 33 patients diagnosed with brief psychosis at 10 different hospitals in Andalusia^21^, concluding that brief psychotic disorder was more common than usual during the first 8 weeks of the lockdown, although they also noted that one-third of these cases had presented similar episodes in the past. The Italian group compared FEP patients admitted during the first four months of the pandemic to those admitted during the same period in 2019, finding a higher incidence of brief psychosis during the pandemic, but no differences between the two periods in the proportion of cases with acute or affective psychosis^22^.

Szmulewicz et al.^23^ compared the characteristics of patients treated at a FEP unit during the first few months of confinement/lockdown (between March and July, 2020) with the corresponding period in 2019. In that study, there were fewer admissions of FEP patients in the 2020 period, but with more severe symptomatology, and a higher proportion of involuntary admissions, potentially indicating a delay in the diagnosis of mild symptomatology.

In short, the COVID-19 pandemic is associated with high psychosocial stress and, consequently, with the development of neuropsychiatric disorders. Psychosocial stressors have been consistently associated with the development of both FEP and brief psychotic disorder. However, the findings of the studies conducted to date are inconsistent, most of which had only a limited follow-up. Furthermore, it is not clear whether FEP diagnosed during the early months of the pandemic is associated with certain sociodemographic or vulnerability factors, nor whether there is a unique clinical presentation. In this regard, it would be valuable to determine the main factors involved in FEP onset and severity in order to develop prevention strategies and to determine how to best mitigate the risks of FEP in future crises or events with similar psychosocial stress characteristics.

In this context, the aim of the present study was to assess the incidence rate (IR) of FEP during the first nine months of the COVID-19 pandemic in Spain and to compare these data to the corresponding period in the prior year. In addition, we explored the clinical and sociodemographic factors associated with FEP onset in these two time periods.

## Methods

### Participants

We compared the sociodemographic and clinical characteristics of patients diagnosed with FEP from March 14, 2020 (the date on which the state of emergency and lockdown was declared in Spain) to December 31, 2020 with those of patients diagnosed with FEP in the same period in the year 2019. We included all patients (both inpatients and outpatients) who presented for the first time with a FEP to one of three specialized psychotic care units (PAE-TPI; “Program of specific care to incipient psychotic disorders”) at the Departments of Psychiatry at the following hospitals in Catalonia, Spain: Parc de Salut Mar (Barcelona, Spain), Parc Taulí (Sabadell, Spain), and the Institut Pere Mata (Reus, Spain). These three departments provide coverage for a catchment area that includes approximately 1.16 million people. The PAE-TPI program is a specialized early intervention service for individuals between the ages of 14 and 35 who experience FEP. It provides a multimodal intervention, including comprehensive clinical evaluation, with intensive medical and psychosocial treatment^24^.

Inclusion criteria for the present study were as follows: (1) age 14–35 years at the time of evaluation; (2) ICD-10^25^ criteria for brief psychotic disorder, schizophrenia, schizoaffective disorder, unspecified psychosis, delusional disorder, mania with psychotic symptoms, depressive episode with psychotic symptoms, (3) duration of untreated psychosis < 12 months; (4) no prior history of severe neurological medical conditions or severe traumatic brain injury; (5) presumed IQ level > 80 based on clinical records (either evidence from past IQ assessments or suggested by the patient’s educational or employment level); (6) no substance abuse or dependence disorders (except for cannabis or nicotine use); (7) no confirmed or suspected SARS-CoV-2 infection (defined as absence of symptoms and no close contact with a confirmed SARS-COV-2 patient). For hospitalized patients, this criterion was confirmed with a negative polymerase chain reaction (PCR) test.

The study was approved by the local Clinical Research Ethics Committee (CEIM) at all three of the participating hospitals. This study is part of a larger study entitled “CEIM 2019/8995-Protocol for clinical care of FEP” originally approved by the CEIM at Parc de Salut Mar. The study was conducted in accordance with all relevant guidelines and legal regulations. All participants provided written informed consent.

### Assessment

#### Sociodemographic and clinical data

The following variables were assessed: age; sex, immigrant status (yes/no); duration of untreated psychosis (DUP), calculated as the number of days between onset of psychotic symptoms and initiation of pharmacological treatment; and cannabis use (yes/no) during the prior three months.

#### Diagnosis

ICD-10 criteria were used for the diagnosis^25^. Patients were grouped into categories based on their ICD-10 diagnostic codes, as follows: brief psychotic disorder (F23) vs. non-brief psychotic disorder (F12.50, F19, F20, F22, F25, F28, F29, F30, F31, F32), and affective (F30, F31, F32) vs. non-affective psychosis (F12.50, F19, F20, F22, F23, F25, F28, F29).

#### Psychopathology

The following symptom scales were administered: the Positive and Negative Syndrome Scale (PANSS) for symptoms related to psychosis^26^; the Young Mania Rating Scale (YMRS)^27^ for manic symptoms; the Calgary Depression Scale for Schizophrenia (CDSS) for depressive symptoms^28^; Global Assessment of Functioning (GAF) to assess functionality^29^. The YMRS, CDSS and GAF scales were not obtained in some patients because some of the PAE-TPI do not routinely administer these scales.

### Statistical analysis

Data distribution normality was assessed with the Kolmogorov–Smirnov test. Population statistics in each area was obtained through the Catalan Institute of Statistics^30^. The IR was calculated as the ratio of new cases/total population*elapsed time from the start of follow-up. IRs were calculated in person-months and rescaled to person-years. Exact Poisson confidence limits for the IRs were derived. Between-period differences in IR were checked from the significance of the IR difference, using the mid-p adjustment to exact p values.

To determine the factors associated with FEP onset in the two time periods, we performed univariate analyses for the sociodemographic/clinical variables and time period of FEP onset. We used Mann–Whitney U test for non-normally distributed continuous data and Chi-squared test for categorical data. Subsequently, a logistic regression analysis was performed using the ENTER method. The dependent variable was the time of FEP onset and the independent variables were the potential vulnerability factors (i.e., immigration age, sex, cannabis use, diagnosis) with a significance level of p ≤ 0.1 on the univariate analysis. The goodness-of-fit was assessed with the Hosmer and Lemeshow test and collinearity was assessed using the variance inflation factor.

All statistical analyses were performed with the IBM-SPSS Statistics for Windows program, v. 20 (IBM Corp.; Armonk, NY, USA). P values ≤ 0.05 were considered statistically significant.

## Results

**Figure 1:**
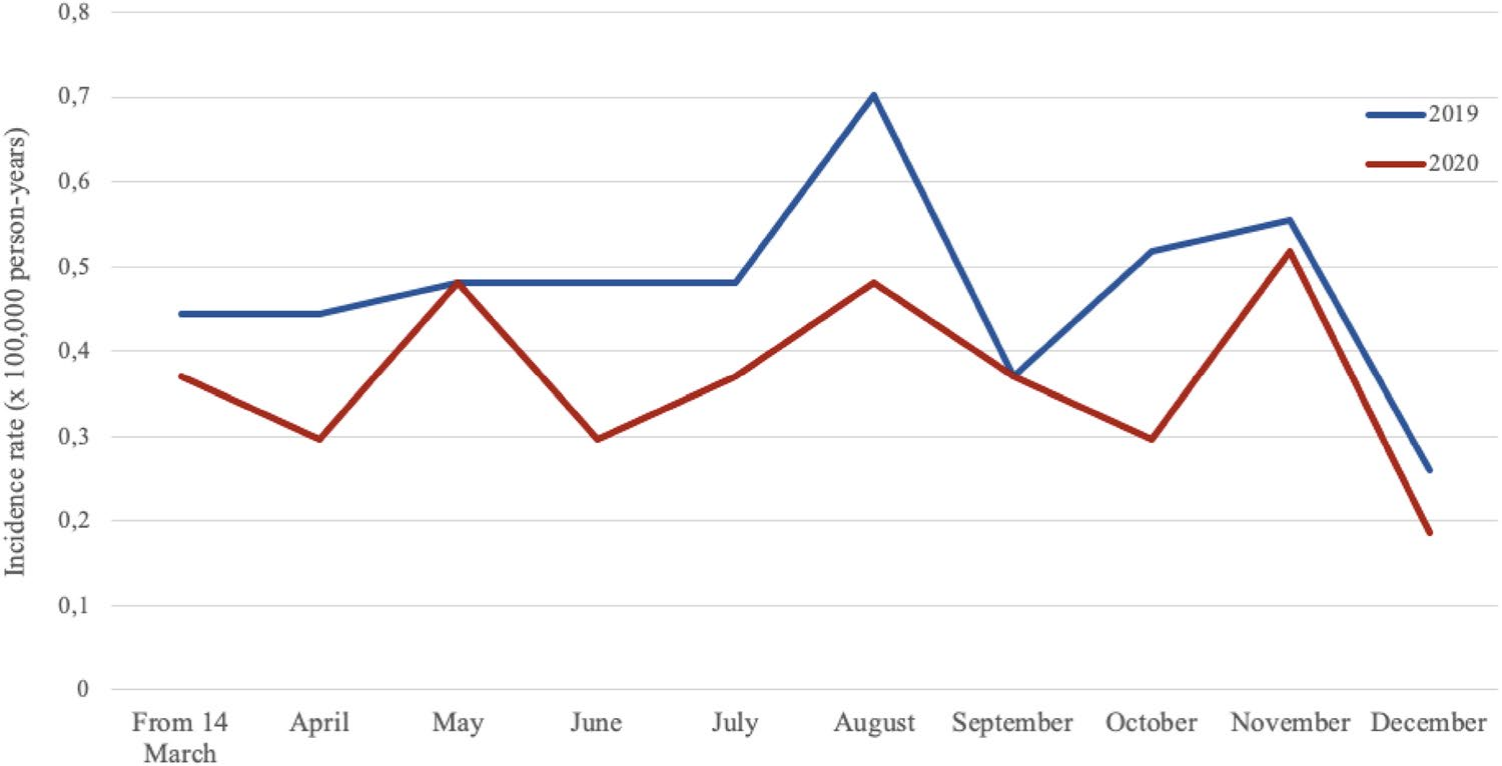
Incidence rate of FEP per month during the COVID-19 pandemic (2020) and the equivalent period in prior year (2019).

A total of 220 patients with FEP were included in this study. Most patients (n = 141; 64.1%) were males. The median age was 23.6 years (interquartile range, 19–27). Most of the sample (n = 135; 61.4%) were diagnosed with FEP during hospital admission. Of the 220 patients, 95 were treated between March 14 and December 31, 2020. The other 125 patients were treated during the corresponding period in 2019. The IR was lower during the COVID-19 pandemic period than in 2019, although this difference did not reach statistical significance (0.42/100,000 person-years vs 0.54/100,000 person-years; p = 0.057). Figure 1 shows the monthly progression of the IR in both time periods.

There were no differences between groups in the proportion of each subtype of psychosis (p = 0.25; Table 1). The proportion of women was higher in the COVID-19 period (46.3% vs 28%; p = 0.005) with a trend towards fewer cannabis users (56.8% vs 68.8%; p = 0.068) compared to the prior year (Table 2). No between-group differences were observed in other sociodemographic variables.

**Table 1:** Diagnosis subtypes of the two groups of FEP patients.

	COVID-19 pandemic period (March 14–Dec 31, 2020), n = 95	Equivalent period in prior year (March 14–Dec 31, 2019), n = 125	Statistic value (χ^2^)	P value
Unspecified psychosis	39 (41.7%)	44 (34.7%)	7.803	0.25
Schizophrenia	30 (31.3%)	37 (29.8%)		
Brief psychotic disorder	10 (10.4%)	10 (8.1%)		
Delusional disorder	1 (1%)	0 (0%)		
Schizoaffective disorder	2 (2.1%)	6 (4.8%)		
Mania with psychotic symptoms	12 (12.5%)	28 (22.6%)		
Depressive episode with psychotic symptoms	1 (1%)	0 (0%)

**Table 2:** Sociodemographic and clinical variables of the two groups of FEP patients.

	COVID-19 pandemic period (March 14–Dec 31, 2020), n = 95*	Equivalent period in the prior year (March 14–Dec 31, 2019), n = 125**	Statistic value (*U* or χ^2^)	P value
Age, median (IQR)	23 (19–27)	23 (19–27)	5914.50	0.96
Gender, N (% woman)	44 (46.3%)	35 (28%)	7.87	**0.005**
Immigrant, N (% immigrant)	45 (47.4%)	47 (37.6%)	2.12	0.15
DUP, median (IQR)	36 (10–120)	30 (14–113.75)	5848.50	0.95
Hospitalized, N (%)	56 (58.95%)	79 (63.2%)	0.41	0.58
Cannabis use, N (% users)	54 (56.8%)	86 (68.8%)	3.34	0.068
Brief psychotic disorder, N (%)	10 (10.5%)	10 (8%)	0.42	0.52
Affective psychosis, N (%)	13 (13.7%)	28 (22.4%)	2.70	0.10
PANSS P score, median (IQR)	30.50 (26–35.75)	32 (27–35)	2664.50	0.95
PANSS N score, median (IQR)	19 (13–28.75)	21 (14–27)	2655.50	0.92
PANSS GP score, median (IQR)	51 (42–58.75)	51 (41–56)	2450	0.37
PANSS T score, median (IQR)	99.50 (84.50–122)	105 (84–116)	2718	0.87
YMRS score, median (IQR)	12 (4–26)	17 (8.5–30.5)	848.50	0.33
CDSS score, median, (IQR)	0 (0–5)	0 (0–4)	944.00	0.84
GAF score, median (IQR)	35 (26–40)	30 (27.5–40)	914.00	0.66

Significant values are in [bold].

*N* sample size, *IQR* interquartile range, *DUP* duration untreated psychosis, *n* sample size, *PANSS P* Positive and Negative Syndrome Scale positive, *PANSS N* Positive and Negative Syndrome Scale negative, *PANSS GP* Positive and Negative Syndrome Scale general pathology, *PANSS T* Positive and Negative Syndrome Scale total, *YMRS* Young Mania Rating Scale, *CDSS* Calgary Depression Scale for Schizophrenia, *GAF* Global Assessment of Functioning.

*PANSS scores were assessed in 67 patients; YMRS, CDS and GAF scores were assessed in 46 patients.

**PANSS scores were assessed in 80 patients; YMRS, CDSS and GAF scores were assessed in 42 patients.

There were no significant between-period differences in the proportion of patients with brief psychotic disorder or affective psychosis. However, there was a non-significant trend (p = 0.07) towards a lower incidence of affective psychosis during the COVID-19 period (13.7% vs 22.4%). There were no significant differences in other clinical variables such as DUP, the proportion of hospitalized patients, or scores on any of the clinical scales.

On the logistic regression analysis, we included sex, cannabis use, and the proportion of patients diagnosed with affective psychosis (Table 3). There was no collinearity among those variables (Variance inflation factor among the variables < 3). The only variable significantly associated with FEP onset in the pandemic period was female sex (Hosmer and Lemeshow test (p = 0.398); B = 0.75; OR 2.12 [CI 1.17–3.82]; p = 0.014).

**Table 3:** Logistic regression analysis to determine the factors associated with FEP onset in the COVID-19 pandemic period vs. the corresponding period in the prior year.

	B	OR	95% CI	P value
Gender	0.75	2.12	1.17–3.82	**0.014**
Cannabis use	− 0.32	0.72	0.40–1.31	0.29
Affective psychosis	− 0.69	0.50	0.24–1.05	0.068
Constant	− 0.22	0.80		0.44

Significant values are in [bold].

*B* coefficient, *OR* odds ratio, *CI* confidence interval.

## Discussion

This study was performed to determine the incidence rate of FEP and to describe the sociodemographic and clinical characteristics of patients diagnosed with FEP during the COVID-19 pandemic (March–December, 2020) compared to patients diagnosed during the same period in the prior year. Our findings show a trend towards a lower IR during the COVID-19 period, but this difference did not reach statistical significance. The only variable significantly associated with a greater risk of FEP during the pandemic period versus the non-pandemic period was female sex.

The similar IR during the pandemic period in our study contrasts with several other studies, which found an increase in the incidence of psychosis after implementation of lockdown measures. For example, Esposito et al.^22^ found a 29.6% increase in FEP patients during the first three months of the pandemic, although it is worth noting that those authors only evaluated hospitalized patients. Segev et al.^31^ found an increased ratio of new-onset psychosis and mania among patients who presented to psychiatric emergency departments in Israel between January and July 2020 compared to the same period of the prior year. Nevertheless, other studies have reported findings that are consistent with our data. For example, Szmulewicz et al.^23^ found a lower admission rate to their FEP program during the first months of lockdown. Nonetheless, it is important to note that those authors included all patients treated in their unit, most of whom were first treated before 2019 (only two cases were enrolled in the program in 2020). Similarly, O’Donoghue et al.^32^ did not find any differences in the overall incidence of FEP at their early intervention service in Melbourne (Australia) during an 8-month period during the COVID-19 pandemic year vs the same period in the prior year. However, as the authors noted, the was an increase in cases in the later months of the study period. In our study, we did not observe a significant increase in the incidence of FEP in the later months of 2020, nor any other visible trend in any particular month of the pandemic (Fig. 1). As O’Donoghue and colleagues observed, it is important to bear in mind that the incidence of COVID-19 was much lower than in Spain, which would explain the differences between the two countries in terms of the effects of the pandemic.

We did not find any increase in the proportion of patients diagnosed with brief psychosis or affective psychosis, two syndromes associated with the affective pathway of psychosis and in which the dominant trigger is stress-related^33^. Some case reports and case series published around the beginning of the pandemic suggested an increase in the number of patients with brief psychotic disorder, potentially attributable to pandemic-related psychosocial stress. Valdés-Florido et al.^21^ described a case series (n = 33) of patients who met criteria for brief psychotic disorder (half of whom also had a past history of psychiatric illness). However, other studies, such as that carried out by Esposito et al.^22^, did not detect any increase in the percentage of FEP patients with brief psychotic disorder in the first four months after lockdown versus the prior year. This finding, which is consistent with our results, suggests that pandemic-related stressors negatively affected patients with a previous history of brief psychotic disorder, but did not significantly increase new cases of brief psychotic disorder.

When we compared the sociodemographic characteristics of patients in the two study periods, we found a higher percentage of women and a trend towards fewer cannabis users in the pandemic period. After controlling for other factors, female sex was the only variable significantly associated with an increased risk of FEP during the pandemic.

Several studies have shown that the pandemic had a greater impact on mental health in women than in men, with higher levels of emotional distress^34^ and an increased risk of PTSD^35^ in women. Apart from the potential influence of neurobiological differences between the two sexes, the sex roles commonly assigned to women in Spanish society may have also placed more stress on women. For example, women probably assumed more responsibility for the care of children and the household during the lockdown, which could have resulted in higher stress levels^36^. However, it is important to note that several studies that evaluated the characteristics of psychotic patients during the pandemic found no differences in the proportion of women with psychosis compared to prior years^21,22,32^. Nonetheless, most of those studies evaluated a shorter time period during the peak of the pandemic, when many patients may have avoided the health care network. In order to minimize this potential effect, we opted to expand the observation period for our study to nine months, thereby including many weeks during which demand for health care due to COVID-19 was lower than in the peak periods, which means mental health resources were more accessible.

Studies have shown that schizophrenia onset occurs later in life and is less severe in women than in men^37^. These sex differences in stress-related disorders have been associated with a protective role of sex hormones, in particular higher levels of estrogen and progesterone^37^. Studies have shown that men who perform social stress tasks in the laboratory have higher cortisol levels than women^38^. By contrast, some researchers have hypothesized that women may have an increased emotional reactivity to stress than men, a hypothesis that is supported by the higher proportion of women with affective psychosis^33^. In our study, the pandemic-related stressors seem to have had a greater negative impact on women, regardless of the specific type of psychosis. In fact, our data show a trend towards fewer cases of affective psychosis during this period. An alternative (or complementary) hypothesis for these sex differences could be that males with FEP tend to consult for behavioral disturbances and negative symptoms^39^, and this tendency may have gone unnoticed during the pandemic because of the general tendency among the population to avoid using health care services during this time period.

In our study, the proportion of FEP patients who reported using cannabis was slightly lower during the COVID-19 pandemic than the prior year, a finding that is consistent with other studies that have observed a decrease in drug abuse comorbidities in patients with FEP^22^. The presence of a comorbid substance abuse disorder (except for cannabis and nicotine) was an exclusion criterion in our study, which means we were not able to compare the two groups in terms of the prevalence substances other than cannabis and nicotine. However, after controlling for other factors, the trend towards lower cannabis use during the pandemic disappeared. In our sample, as in other studies^40^, a significantly lower percentage of women used cannabis (44.3% vs. 74.5% in men; p < 0.001). Consequently, the lower rates of cannabis use in our full cohort during the pandemic could be attributed to the higher proportion of women in the sample. During the strict home confinement period, a decrease in drug use should be expected due to the difficulties of buying illegal drugs, although (somewhat surprisingly) some studies actually found an increase in cannabis and alcohol use, especially among men^41^. In a large sample of cannabis users in Netherlands, most daily users increased their use after the lockdown while non-daily users also increased the frequency of cannabis use^42^. This increase in cannabis use in the general population during the pandemic does not appear to be any associated with a higher risk of developing FEP, which further strengthens the hypothesis that patients who developed FEP during the pandemic period had a different pattern of vulnerability to known risk factors for psychosis.

We observed a non-significant trend towards a higher percentage of immigrants among the FEP patients during the pandemic period. Studies show that first- and second-generation immigrants have a higher risk of psychosis, perhaps due to the perception of social inequality^43^. In the context of the COVID-19 crisis, these inequalities may have been exacerbated in the most vulnerable populations, such as immigrants. Future studies are warranted to better understand the relationship between immigration and FEP during the pandemic.

In line with previous reports^22^, we observed no differences in psychopathological scores among the individuals who developed FEP during the pandemic versus the prior year, suggesting that the clinical presentation was no more severe than usual. Other studies have suggested that, due to the pandemic-related restrictions, FEP patients may have presented more severe symptoms because they delayed contacting their healthcare provider, which implies a delay in treatment initiation^23,32^. O’Donoghue et al.^32^ found an increase in the proportion of admitted FEP patients during the COVID-19 pandemic period, which they attributed to a possible increase in symptom severity at diagnosis and changes in how the clinical unit delivered treatment. Contrary to expectations, we did not detect any increase in the DUP or the proportion of admitted patients, which suggests that the FEP assistance network in our area maintained its normal functioning despite the restrictions.

We found no differences between the two time periods in terms of mean patient age, a finding that contrasts with previous studies that have reported a higher mean age for psychotic episodes diagnosed after implementation of lockdown measures. For example, in the study by Esposito et al.^22^, the mean age in 2019 was 34.0 years vs. 43.5 years in 2020 (with many patients over age 50). The initial debut of a psychotic episode in patients over age 40 is highly suggestive of the presence of an affective disorder or an incipient neurodegenerative disorder; nevertheless, Esposito and colleagues did not observe a higher proportion of patients with affective psychosis^22^. In our study, we only included patients ≤ age 35 since this is the age limit for FEP programs in our area. Consequently, we have no data on late onset cases.

### Strengths and limitations

The present study has several limitations. First, most of the outpatients did not take a PCR test since this test was only ordered for symptomatic or hospitalized patients. Therefore, we cannot rule out the presence of an asymptomatic COVID-19 infection in those patients. Although SARS-CoV-2 infection mainly affects the respiratory system, it can also be accompanied by neuropsychiatric symptoms^4^. Psychotic symptoms can occur with delirium in severe COVID-19, but have also been described as primary psychotic episodes in mild infections, even when asymptomatic^44–46^. Consequently, we cannot rule out the possibility that some of the FEP cases in our sample were secondary to the virus or the associated hyperinflammatory state. However, previous studies suggest that the percentage of COVID-19 infected patients among FEP patients is very low^31^. Another limitation is that the psychotic symptoms in some FEP patients may have begun before the lockdown measures were implemented, which would affect the DUP, which is why we used a more objective measure of FEP onset: the date of first contact with the FEP program. Another limitation is that our sample included patients from three different regions in Catalonia, each with its own FEP program; as a consequence, there were inter-program differences in the assessment protocols and thus some variation in the assessment tools. As a result, we were unable to assess some of the scales in a small subgroup of patients. Another limitation is that we evaluated cannabis use through a clinical interview with the patient rather than by a more objective (i.e., quantitative) method, which would have given us more precise data. That said, this type of assessment was not feasible due to the pandemic, which is why most similar studies have also used subjective methods^41,42^.

Despite these limitations, this study has several important strengths. First, we evaluated the incidence rate for FEP in a large geographic area encompassing > one million inhabitants, including 300,000 people between the ages of 16 and 35. Another strength is that all three participating FEP programs were established many years ago, with highly experienced staff and well-established protocols. These programs are the primary treatment option for FEP patients, offering multidisciplinary care. Consequently, our sample included the vast majority of FEP cases treated in our health care system. Furthermore, these programs have not undergone any major modifications in recent years, which ensures that the differences observed between the two study periods can be attributed to the pandemic rather than to changes in the FEP program.

## Conclusions

In conclusion, our data show a trend towards a lower incidence of FEP during the pandemic when compared to the same time period in the prior year. The only variable associated with a greater risk of developing FEP during the pandemic versus the non-pandemic period was female sex. In future studies, it would be useful to determine the course of psychosis among the patients who developed FEP during the pandemic in order to determine whether the clinical trajectory in these patients differs from patient cohorts in other time periods.

## Data Availability

The data that support the findings of this study are available from the corresponding author, Anna Mané, upon reasonable request.
